# Modern Aspects of Diabetes

**Published:** 1893-10-07

**Authors:** 


### Modern Aspects of Diabetes.

Probably there is no disease with a wider literature than diabetes. Both to the clinical physician and to the experimentalist it has always been a most fascinating study, and the more observant physicians have taught for some time past that there are many diseases which at present we cannot satisfactorily separate, but in all of which glycosuria is a prominent symptom, just as there are many maladies all of which give rise to the presence of albumen in the urine. Morbid anatomists have often been struck with the fact that in some patients dying of diabetes the pancreas has been found to be diseased, and this has become particularly interesting, as it has been discovered that diabetes can be experimentally produced by excising this organ in certain animals, especially cats, dogs, pigs, and carnivorous birds, although, strange to say, not in vegetable-feeding birds, as ducks and pigeons. Now the cardinal fact to grasp is that never before has diabetes been produced experimentally. Puncture of the floor of the fourth ventricle, section of the splanchnic nerves, and the numerous other operations on nerves, which are described in the physiology books as causing diabetes, strictly speaking only produce glycosuria, probably because they lead to a dilatation of the hepatic vessels, as a result of which the sugar conveyed to the liver by the portal vein does not rest in the liver long enough to be stored up as glycogen, but is hurried on into the general circulation and excreted in the urine. This glycosuria is transient, and does not impair the health of the animal. It is exactly comparable to the glycosuria seen in elderly people who overeat themselves, and to the glycosuria which may often be met with as a result of shock or worry. As it is well known these forms in man are not particularly harmful, and easily respond to simple treatment.

Diabetes, however, is a very different affair; in addition to the glycosuria we have many other symptoms, such as wasting and coma, and, as just mentioned, the experimentalist has at last succeeded in causing diabetes in animals, for when the pancreas of a dog, for example, is excised, the creature dies in a few days from rapid and severe diabetes. This is a striking contrast to the results that would have followed puncture of the floor of the fourth ventricle in the same animal, all that would then have been noticed would have been transient glycosuria. Minkowski's recent paper ("Arch. f. exp. Pathol. u. Pharmakol," vol. xxxi., pp. 85-189) is one of the most elaborate and perfect papers in experimental medicine, and he treats the whole subject of diabetes after extirpation of the pancreas in a most exhaustive manner, and shows that in the case of dogs the sugar appears in the urine the very day after the excision of the pancreas, and attains its maximum on the third day, after which, as in human diabetes so in dogs, the amount of sugar in the urine varies directly according to the diet given. It is well-known that in human diabetes the most rigorous restriction of diet will not, if the case is severe, lead to the total absence of sugar in the urine; so it is with dogs, they use up the albuminous tissues of the body, as is shown by the excessive wasting, and one of the products of this breaking down of tissues is the sugar which appears in the urine, quite irrespective of the diet. Another point in which dogs resemble man is that as death approaches the sugar in the urine lessens.

Two or three years ago, early in the history of the research on pancreatic diabetes, the remarkable fact was discovered that if a part only of the pancreas is taken out no diabetes is observed. The precise proportion which must be left varies, but as a rule, if about a fifth of the organ remains, diabetes does not follow upon the excision of the other four-fifths. Such a result paves the way for the next extraordinary fact we have to record. Hédon ("Arch. de Phys.," Oct., 1892) has shown—and Minkowski agrees with him—that if the pancreas (or, for the matter of that, a large piece of it) be excised from all its connections, except that it is left attached to its main vessels, and that if then it be transplanted under the skin of the abdomen it will not perish. After a few days new vessels from the surrounding subcutaneous tissue enter it, and then the original pancreatic vessels may be cut without damaging the transplanted organ. No diabetes results. Directly, however, the transplanted organ is excised or its new vessels are ligatured, so that it undergoes necrosis, fatal diabetes is induced. Clearly we have reached a very important conclusion, namely, that the diabetes brought about by excision of the pancreas depends upon the fact that there is a specific function of this organ which is not exerted through its secretion, and therefore is almost certainly exerted on the blood circulating through it, and that so long as about a fifth of the organ is left, there is enough of it to carry on this function, in virtue of which sugar does not appear in the blood and urine, and the other symptoms of diabetes do not show themselves, but as soon as a larger amount of the gland is excised or destroyed there is not sufficient pancreas to carry on this function, the withdrawal of which leads to diabetes. After this we are not surprised to find that neither cutting the nerves going to the organ, nor ligature of its duct leads to diabetes.

It is a striking fact that in man so little of importance is found after death from diabetes, for the enlargement of the kidneys is probably only due to the large amount of work they have to do. This quite accords with the fact that Minkowski was unable to find that excision of other organs than the pancreas would cause diabetes. Thus he was not able to confirm the results of those workers who stated that diabetes follows after excision of the salivary glands, thyroid, or duodenum. Nor is this surprising, for if there were other organs having this function as well as the pancreas the difference between excising four-fifths and, say, nineteen-twentieths ought not to be as marked as it is. Bearing in mind the similarity of this pancreatic diabetes with many severe human cases, and the fact that in man the pancreas is often the only organ found to be abnormal at the autopsy, it is highly probable that some cases of severe diabetes in man are due to disordered function of the pancreas. It is not against this view that malignant disease of the pancreas does not induce diabetes, for we know that malignant disease of the kidney does not cause Bright's Disease, nor does malignant disease of the thyroid induce myxœdema.

As injecting extract of thyroid under the skin or feeding on thyroid cures myxœdema, it naturally suggested itself that feeding on fresh, raw pancreas might benefit diabetes. Capparelli has, indeed, stated that injecting pancreas from healthy dogs into those whose pancreas has been excised prevents diabetes, but Gley and others have adversely criticised these results, and some cases published by Hale White showed that feeding patients suffering from diabetes upon raw pancreas does not in any way modify the disease.

